# Robotic Versus Open Radical Hysterectomy in Early-Stage Cervical Cancer: A Comparative Cohort Study

**DOI:** 10.3390/jcm15135168

**Published:** 2026-07-02

**Authors:** Anna Jędrzejczyk, Krzysztof Mawlichanów, Agnieszka Golec-Cera, Marcin Opławski

**Affiliations:** 1 Department of Gynecology, NEO Hospital, 30-437 Kracow, Poland; anna@jedrzejczyk.com.pl (A.J.); mawlichanow@gmail.com (K.M.); agagoleccera@gmail.com (A.G.-C.); 2 Department of Gynecology and Obstetrics, Faculty of Medicine and Health Sciences, Andrzej Frycz Modrzewski Krakow University, 30-705 Krakow, Poland; 3 Department of Gynecology and Obstetrics with Gynecologic Oncology, Ludwik Rydygier Memorial Specialized Hospital, 31-820 Kracow, Poland; 4 Bioethics and Medical Law Department, Andrzej Frycz Modrzewski Kraków University, 30-705 Kracow, Poland

**Keywords:** cervical cancer, robotic surgery, radical hysterectomy, total mesometrial resection, minimally invasive surgery

## Abstract

**Background/Objectives:** Following the LACC trial, the role of minimally invasive radical surgery for early-stage cervical cancer remains controversial. Robotic-assisted approaches have been proposed as a potential strategy to preserve the benefits of minimally invasive surgery while incorporating contemporary oncologic precautions. This study compared perioperative, pathological, and early oncologic outcomes between robotic and open radical surgical management in patients with FIGO 2018 stage IA2–IIA1 cervical cancer. **Methods:** Patients underwent robotic surgery (n = 20; da Vinci Xi), including robotic radical hysterectomy, compartment-based procedures, and fertility-sparing surgery when clinically indicated, or open abdominal radical hysterectomy (n = 22). Perioperative outcomes, histopathological parameters (including lymphovascular space invasion [LVSI], lymph node status, and margin status), and early oncologic outcomes were evaluated. Exploratory multivariable regression analyses were performed to adjust for baseline differences, including age and tumor size. **Results:** Patients in the open-surgery cohort were older (56.23 ± 15.87 vs. 45.67 ± 9.31 years; *p* = 0.012) and had significantly larger tumors (3.07 ± 1.10 vs. 1.4 ± 0.7 cm; *p* = 0.003). Robotic surgery was associated with longer operative time (178 ± 42 vs. 150 ± 38 min; *p* = 0.028), lower blood loss (112 ± 61 vs. 518 ± 98 mL; *p* < 0.001), and shorter hospital stay (4.2 ± 1.6 vs. 6.2 ± 1.4 days; *p* < 0.001). The robotic cohort also demonstrated a higher lymph node yield (median 18 vs. 9; *p* < 0.001). No statistically significant differences were observed between groups in lymph node metastasis (20.0% vs. 22.7%; *p* = 1.000), LVSI (33.3% vs. 63.6%; *p* = 0.121), or R0 resection rate (100% vs. 95.5%; *p* = 1.000). In exploratory adjusted analyses, surgical approach was not associated with adverse pathological features, whereas tumor size emerged as an independent predictor of both lymph node metastasis and LVSI. No recurrences were observed in the robotic cohort during the available follow-up period. **Conclusions:** In this exploratory comparative cohort study, robotic radical surgical management in carefully selected patients with predominantly small-volume disease was associated with favorable perioperative outcomes and no statistically significant differences in pathological parameters compared with open surgery. Tumor size, rather than surgical approach, emerged as the principal predictor of adverse pathological features. Given the limited sample size, baseline imbalances between cohorts, heterogeneous robotic procedures, and absence of mature survival data, these findings should not be interpreted as evidence of oncologic equivalence and require confirmation in larger prospective studies.

## 1. Introduction

Despite considerable progress in preventive strategies, including population-based screening programs, prophylactic HPV vaccination, and improvements in early diagnostic methods, cervical cancer continues to represent a major global health burden and remains one of the leading causes of cancer-related morbidity and mortality among women worldwide [1,2]. According to contemporary recommendations issued by the European Society of Gynaecological Oncology (ESGO), the European Society for Radiotherapy and Oncology (ESTRO), the European Society of Pathology (ESP), and the Polish Society of Gynecologic Oncology (PTGO), surgery remains the preferred treatment modality for most patients with early-stage disease up to FIGO stage IIA1 [3,4]. Exceptions include patients with FIGO IB2 and IIA2 tumors, for whom definitive chemoradiotherapy is generally recommended. Management of microinvasive disease is determined by the extent of invasion; patients with FIGO IA1 tumors can often be treated conservatively using conization or simple hysterectomy, whereas FIGO IA2–IIA1 disease typically requires radical hysterectomy combined with pelvic lymph node assessment. Regardless of the surgical technique employed, the principal oncologic objective is complete tumor removal with histologically negative margins, thereby minimizing the risk of residual disease and recurrence.

Although FIGO staging remains fundamental for treatment planning, accumulating evidence suggests that clinical outcomes are strongly influenced by intrinsic tumor biology. Established pathological risk factors, including primary tumor size, depth of cervical stromal infiltration, lymphovascular space invasion (LVSI), and lymph node involvement, significantly affect the probability of recurrence and disease progression. Furthermore, molecular and genetic characteristics of the tumor increasingly contribute to risk stratification and may partially explain the heterogeneity of treatment responses observed among patients with apparently similar clinicopathological features. Persistent high-risk human papillomavirus infection initiates malignant transformation [5,6]; however, subsequent progression is driven by complex interactions involving epithelial–mesenchymal transition, angiogenesis, immune evasion, genomic instability, and activation of signaling pathways such as PI3K/AKT/mTOR, TGF-β, Wnt/β-catenin, and hypoxia-associated networks. These biological processes contribute to metastatic dissemination and may ultimately exert a greater influence on long-term oncologic outcomes than the surgical approach itself. Consequently, contemporary evaluation of surgical strategies should be considered within the broader context of tumor biology and recurrence risk [7,8,9].

Advances in diagnostic imaging, particularly pelvic magnetic resonance imaging (MRI), have significantly improved preoperative staging accuracy and patient selection, gradually replacing earlier diagnostic strategies based primarily on clinical examination and urography [10,11]. Despite ongoing technological progress, open abdominal radical hysterectomy has traditionally been regarded as the reference standard, providing reliable oncologic radicality through wide parametrial and paravaginal resection.

The introduction of minimally invasive surgical techniques, including laparoscopy and robotic surgery, initially transformed the surgical management of cervical cancer by reducing perioperative morbidity, blood loss, and length of hospitalization while maintaining surgical precision [12]. However, the publication of the landmark Laparoscopic Approach to Cervical Cancer (LACC) trial fundamentally altered clinical practice by demonstrating inferior disease-free and overall survival associated with minimally invasive radical hysterectomy compared with open surgery [13]. These findings led to a global reassessment of minimally invasive approaches and a widespread return to open surgery as the preferred modality for early-stage cervical cancer [14,15].

Subsequent studies and meta-analyses have explored potential explanations for these findings and attempted to refine patient selection and surgical techniques [16,17]. Factors such as surgical technique, tumor manipulation, surgeon experience, institutional volume, and strict adherence to oncologic principles have been proposed as critical determinants of outcomes, potentially outweighing the influence of the surgical approach itself. In this context, compartment-based surgical concepts, including total mesometrial resection (TMMR) derived from the ontogenetic cancer field theory [18], have emerged as a biologically grounded strategy aimed at enhancing oncologic radicality while preserving functional outcomes [19,20].

Robotic technology offers distinct advantages for the implementation of complex pelvic oncologic procedures, including enhanced three-dimensional visualization, tremor filtration, and improved instrument articulation, which may facilitate precise dissection within embryologically defined anatomical planes [21]. These characteristics have stimulated growing interest in robotic-assisted radical surgery and compartment-based approaches in the post-LACC era. Nevertheless, robust comparative evidence evaluating robotic radical surgery in relation to open radical hysterectomy remains limited, particularly in carefully selected patients with early-stage cervical cancer [22,23,24].

Against this background, further evaluation of robotic surgical management is warranted to better characterize its perioperative performance and pathological outcomes within contemporary clinical practice. Therefore, in the post-LACC era, we aimed to compare perioperative, pathological, and early oncologic outcomes between robotic radical surgical management performed with contemporary oncologic precautions and open radical hysterectomy in patients with FIGO 2018 stage IA2–IIA1 cervical cancer [25,26,27].

Given the exploratory nature of the study and the non-randomized design, the objective was not to establish oncologic equivalence but rather to evaluate the feasibility and clinical performance of robotic radical surgery in a real-world setting.

## 2. Materials and Methods

### 2.1. Study Design

This study was conducted as a comparative cohort analysis evaluating perioperative, histopathological, and early oncologic outcomes in patients with early-stage cervical cancer treated with robotic or open radical surgery. The robotic cohort was derived from a previously published prospective pilot study investigating robotic radical surgery, including compartment-based procedures such as total mesometrial resection (TMMR) [23,28]. The comparator cohort consisted of retrospectively identified patients treated with conventional open radical hysterectomy during an earlier treatment period.

The present investigation expands the previously reported robotic experience by incorporating an open-surgery comparator group and additional statistical analyses, thereby enabling direct evaluation of surgical approaches in early-stage cervical cancer. To maximize comparability, definitions of clinical variables, surgical procedures, pathological parameters, and outcome measures were harmonized across cohorts.

The study was conducted in accordance with the Declaration of Helsinki and was approved by the Bioethics Committee of Andrzej Frycz Modrzewski Krakow University, Poland (approval no. KBKA/47/0/2020, issued 9 December 2020). Written informed consent was obtained from patients enrolled in the prospective robotic cohort. Data from the retrospective open-surgery cohort were anonymized before analysis.

### 2.2. Patient Population and Cohort Characteristics

Patients with histologically confirmed early-stage cervical cancer treated with primary surgery were eligible for inclusion. All cases were classified according to the FIGO 2018 staging system as stage IA2–IIA1. Preoperative qualification for radical surgery was based on a standardized diagnostic pathway including gynecological examination, colposcopy, histopathological verification by biopsy or conization, and radiological assessment. The robotic cohort consisted of patients treated at the Department of Gynecology, NeoHospital, Cracow, Poland, between 2021 and 2023. The open-surgery cohort comprised patients treated at the Department of Gynecology and Obstetrics with Gynecologic Oncology, Ludwik Rydygier Memorial Specialized Hospital, Cracow, Poland, between 2018 and 2021. In selected cases, particularly within the IA2 subgroup, final staging was confirmed following postoperative histopathological examination. Surgical decision-making, however, was based on preoperative clinical and radiological assessment. Two surgical cohorts were analyzed. The robotic cohort included patients treated using the da Vinci Xi surgical system. Specifically, 12 patients underwent robotic TMMR, 4 underwent robotic radical hysterectomy, and 4 underwent robotic radical trachelectomy. Because of the limited number of patients within individual procedural subgroups, separate subgroup analyses were not performed. Surgical management was individualized according to tumor characteristics, fertility-preservation considerations, and surgeon judgment. Consequently, the robotic cohort should be interpreted as representing a spectrum of robotic radical surgical approaches rather than a homogeneous TMMR-treated population.

The open-surgery cohort included patients who underwent conventional abdominal radical hysterectomy with pelvic lymphadenectomy via laparotomy. All open procedures were performed according to established oncologic standards for the surgical management of early-stage cervical cancer.

Exclusion criteria comprised FIGO stage above IIA1, recurrent disease, metastatic disease at diagnosis, prior pelvic radiotherapy, prior chemotherapy, neoadjuvant treatment, incomplete clinical or pathological data, and cases in which the preoperative diagnostic assessment was not fully comparable with the standardized staging protocol used in the study cohorts.

The observed differences in baseline characteristics between cohorts reflect the non-randomized nature of patient selection and changes in clinical practice over time. The open-surgery cohort was treated during an earlier period (2018–2021), when radical hysterectomy remained routinely performed in patients with tumors up to 4 cm, consistent with prevailing standards before widespread implementation of post-LACC recommendations. In contrast, patients undergoing robotic surgery were treated between 2021 and 2023 and were selected according to more restrictive criteria, with greater emphasis on smaller tumor burden and favorable preoperative characteristics. Consequently, the robotic cohort included younger patients and patients with significantly smaller tumors. These differences should be considered when interpreting comparative outcomes, as they introduce potential selection bias and limit direct comparability between groups.

Baseline demographic and clinicopathological characteristics, including age, body mass index, tumor size, FIGO stage, and histological subtype, are presented in Table 1. Postoperative management, including duration of hospitalization and perioperative care, followed institutional protocols applied consistently within each cohort.

### 2.3. Preoperative Assessment

All patients underwent standardized preoperative evaluation consisting of gynecological examination, colposcopy, and histopathological confirmation of the cervical lesion obtained by biopsy or diagnostic conization. Radiological assessment was performed to determine tumor extent and suitability for surgical treatment. Pelvic magnetic resonance imaging (MRI) served as the primary imaging modality for assessment of tumor size, stromal invasion, parametrial involvement, and regional lymph node status. When clinically indicated, a transvaginal or transrectal ultrasound performed by experienced sonographers was used to complement MRI findings. Imaging studies were used to determine tumor extent and suitability for surgical treatment.

Additional preoperative investigations included chest imaging to exclude distant metastatic disease, as well as routine laboratory testing comprising complete blood count, serum biochemistry, and coagulation parameters.

Clinical and demographic variables, including age, body mass index, tumor size, histological subtype, and FIGO stage, were systematically recorded.

### 2.4. Surgical Procedures

Robotic procedures were performed using the da Vinci Xi surgical system by board-certified gynecologic oncologists trained in robotic pelvic surgery and compartment-based oncologic techniques. The operative approach followed the principles of radical pelvic oncologic surgery and included robotic radical hysterectomy, TMMR, or radical trachelectomy according to individual clinical indications, tumor characteristics, and fertility-preservation considerations.

The robotic surgical technique incorporated systematic pelvic lymphadenectomy, ureteral dissection, radical resection of paracervical and paravaginal tissues, and nerve-sparing procedures when oncologically appropriate. Particular attention was paid to minimizing tumor manipulation and reducing the risk of intraperitoneal tumor exposure. Measures included avoidance of conventional transuterine uterine manipulators, limited direct handling of the cervix and tumor-bearing tissue, maintenance of dissection within oncologically appropriate anatomical planes, early vascular control when feasible, specimen containment during extraction, and avoidance of tumor exposure during colpotomy. When vaginal transection was required, every effort was made to maintain adequate oncologic margins while minimizing contact between tumor tissue and the peritoneal cavity. In addition, a MyTube^®^ vacuum retractor (AMI—Agency for Medical Innovations GmbH, Feldkirch, Austria) was routinely employed during robotic procedures. Unlike conventional transuterine uterine manipulators, the device does not enter the uterine cavity and does not traverse the endocervical canal. Cervical stabilization is achieved through vacuum-assisted attachment to the ectocervical surface, facilitating atraumatic handling while avoiding intracavitary instrumentation. The device was used to improve surgical exposure while minimizing direct manipulation of the tumor-bearing cervix. The use of this system was intended to facilitate atraumatic cervical stabilization while avoiding intracavitary instrumentation and minimizing manipulation of the tumor-bearing cervix.

The robotic protocol was consistent with the methodology described in the previously published prospective study [23,28], with minor adaptations necessary for comparative analysis.

Open surgery was performed through a midline laparotomy by experienced gynecologic oncologists according to established oncologic principles. The procedure consisted of radical hysterectomy with parametrial and paravaginal resection combined with systematic pelvic lymphadenectomy. The extent of radicality corresponded to Querleu–Morrow type C1 surgery and was individualized according to tumor characteristics and anatomical considerations [29].

Pelvic lymphadenectomy was performed according to institutional standards and included removal of lymphatic tissue from the external iliac, internal iliac, obturator, and common iliac nodal regions. Sentinel lymph node mapping was not routinely employed during the study period. Para-aortic lymphadenectomy was performed only in selected patients based on clinical indications and was not included in the comparative analysis of pelvic lymph node yield. Tumor size was determined from the final histopathological examination in both study cohorts. The largest tumor dimension reported in the final pathology report was used for all comparative and statistical analyses. Histopathological assessment was performed according to institutional pathology protocols and served as the reference source for tumor-size evaluation.

#### Surgeon Experience and Institutional Setting

All procedures were performed by board-certified gynecologic oncologists with substantial experience in radical pelvic surgery. Robotic procedures were performed at NeoHospital, Cracow, Poland, by surgeons trained in robotic gynecologic oncology and compartment-based surgical techniques, including total mesometrial resection. Open procedures were performed at the Department of Gynecology and Obstetrics with Gynecologic Oncology, Ludwik Rydygier Memorial Specialized Hospital, Cracow, Poland, by experienced gynecologic oncologists routinely performing radical hysterectomy for cervical cancer.

Detailed surgeon-volume metrics, including the cumulative number of robotic radical hysterectomies, laparoscopic radical hysterectomies, open radical hysterectomies, and compartment-based procedures performed before the study period, were not systematically available for retrospective analysis. Because the cohorts originated from different institutions and were treated by different surgical teams, surgeon-related and institution-related factors may have influenced perioperative and pathological outcomes independently of surgical approach. Consequently, the independent effects of surgical approach, surgeon expertise, and institutional practice patterns could not be disentangled. All robotic procedures were performed by board-certified gynecologic oncologists formally certified in robotic surgery. One of the two operating surgeons held proctor certification, while the other was an experienced, certified robotic surgeon. This high level of surgical expertise is particularly relevant in the context of contemporary evidence suggesting that oncologic outcomes following minimally invasive radical surgery may be influenced not only by surgical approach but also by surgeon experience, institutional volume, adherence to oncologic principles, and procedural quality. Findings from post-hoc analyses of the LACC trial, as well as emerging data from the RACC study, indicate that outcomes may vary substantially across centers and surgical teams, emphasizing the importance of specialized training and standardized operative techniques.

### 2.5. Histopathological Evaluation

All surgical specimens underwent routine histopathological examination by specialized gynecologic pathologists according to institutional protocols. Histopathological assessment included tumor size, histological subtype, tumor grade, depth of stromal invasion, lymphovascular space invasion (LVSI), parametrial involvement, vaginal margin status, surgical margin status, and lymph node involvement. Lymph nodes retrieved during pelvic lymphadenectomy were examined for metastatic disease. Resection margins were classified as negative (R0) or microscopically positive (R1) according to standard pathological criteria. Histopathological findings were subsequently used to determine pathological risk factors and guide recommendations regarding adjuvant treatment.

### 2.6. Outcome Measures and Follow-Up

The primary objective of the study was to compare perioperative, pathological, and early oncologic outcomes between robotic and open radical surgical approaches in patients with early-stage cervical cancer.

Primary endpoints included lymph node metastasis, lymphovascular space invasion (LVSI), surgical margin status, recurrence, and disease-free survival. Secondary endpoints comprised operative time, estimated blood loss, length of hospital stay, and perioperative complications.

Postoperative complications were classified according to the Clavien–Dindo system [30,31]. Patients were followed according to institutional protocols and contemporary clinical guidelines, including scheduled outpatient visits with gynecological examination, cytological assessment, and imaging studies when clinically indicated.

Follow-up duration differed between cohorts. The robotic cohort was derived from a prospective pilot study with a predefined follow-up period and had a median follow-up of 24 months (range: 24–30 months). The open-surgery cohort had a median follow-up of 60 months (range: 24–96 months), reflecting its retrospective design and earlier treatment period (2018–2021).

Adjuvant treatment was administered selectively based on established pathological risk factors, including lymph node involvement, margin status, parametrial invasion, LVSI, tumor size, and other features considered clinically relevant by the multidisciplinary treatment team.

Recurrence was defined as radiologically or histologically confirmed local, regional, or distant disease.

### 2.7. Statistical Analysis

Data analysis was conducted using StatPlus version 1.1 (AnalystSoft Inc., Brandon, FL, USA). Quantitative variables are reported as either mean ± standard deviation or median with corresponding ranges, depending on their distributional characteristics, whereas qualitative variables are summarized as frequencies and percentages. The distribution of continuous data was evaluated using the Shapiro–Wilk normality test. Differences between the robotic and open-surgery groups were assessed according to data type and distribution. For continuous variables demonstrating normal distribution, comparisons were performed using Student’s *t*-test, while the Mann–Whitney U test was applied to variables that did not meet normality assumptions. Due to the relatively small cohort size and low frequency of several categorical outcomes, Fisher’s exact test was used for comparisons of categorical data. To explore potential predictors of lymph node metastasis and LVSI, multivariable logistic regression models were constructed. Covariates were selected based on their clinical relevance and observed baseline differences between study groups. Owing to the limited number of patients and outcome events, these regression analyses were considered exploratory in nature and intended primarily for hypothesis generation. Therefore, the resulting odds ratio estimates should be interpreted cautiously because of the potential for reduced model stability. Survival analyses using the Kaplan–Meier methodology were not undertaken because follow-up duration differed substantially between cohorts, and no recurrence events were documented in the robotic-surgery group. All statistical analyses were performed using two-tailed tests, and statistical significance was defined as a *p*-value below 0.05.

## 3. Results

### 3.1. Study Population and Baseline Characteristics

The study included 42 patients, of whom 20 underwent robotic surgery, and 22 underwent open radical hysterectomy via laparotomy. Patients in the robotic cohort were significantly younger than those in the open-surgery cohort (45.67 ± 9.31 vs. 56.23 ± 15.87 years, *p* = 0.012). No significant differences were observed in anthropometric characteristics, including body weight, height, and body mass index, although a trend toward higher BMI values was noted in the open-surgery group.

The prevalence of HPV infection was higher in the open-surgery cohort (36.36% vs. 20.0%), whereas HSV infection was infrequent in both groups; however, neither difference reached statistical significance (*p* = 0.28 and *p* = 0.59, respectively). Colposcopy was performed in all patients. Diagnostic conization before definitive surgery was more frequently performed in the robotic cohort (40.0% vs. 13.6%), although the difference did not reach statistical significance (*p* = 0.08).

Tumor size differed significantly between groups, with smaller lesions observed in the robotic cohort (1.4 ± 0.7 cm vs. 3.07 ± 1.1 cm, *p* = 0.003).

Overall, most baseline variables were comparable between cohorts. However, significant differences in patient age and tumor size were observed, indicating that the groups were not fully balanced at baseline. These differences should be considered when interpreting subsequent comparisons of perioperative, pathological, and oncologic outcomes.

### 3.2. Perioperative Outcomes

Comparative analysis of perioperative and pathological outcomes revealed several statistically significant differences between robotic and open surgical approaches (Table 2).

Tumors in the robotic cohort were significantly smaller than those in the open-surgery cohort (1.4 ± 0.7 cm vs. 3.07 ± 1.1 cm, *p* = 0.003). The robotic cohort also demonstrated a higher number of retrieved lymph nodes (median 18 vs. 9, *p* < 0.001).

The incidence of lymph node metastases was comparable between groups (20.0% vs. 22.7%, *p* = 1.000). Similarly, lymphovascular space invasion (LVSI) was observed more frequently in the open-surgery cohort (63.6% vs. 33.3%), although the difference did not reach statistical significance (*p* = 0.121). Complete (R0) resection was achieved in all patients undergoing robotic surgery and in 95.5% of patients treated with open surgery (*p* = 1.000).

With respect to perioperative outcomes, robotic procedures were associated with a longer operative time than open surgery (178 ± 42 vs. 150 ± 38 min, *p* = 0.028). However, robotic surgery was also associated with significantly lower estimated blood loss (112 ± 61 vs. 518 ± 98 mL, *p* < 0.001) and a shorter duration of hospitalization (4.2 ± 1.60 vs. 6.2 ± 1.40 days, *p* < 0.001).

### 3.3. Histopathological and Oncologic Outcomes

Histopathological outcomes differed between cohorts (Table 2). Lymphovascular space invasion was detected more frequently in the open-surgery cohort than in the robotic cohort (63.6% vs. 33.3%), although the difference did not reach statistical significance (*p* = 0.121, Fisher’s exact test). The incidence of lymph node metastases was comparable between groups (22.7% vs. 20.0%; *p* = 1.000, Fisher’s exact test).

Complete (R0) resection was achieved in all robotic cases, whereas one patient (4.5%) in the open-surgery cohort had microscopically positive margins. This difference was not statistically significant (*p* = 1.000, Fisher’s exact test).

### 3.4. Exploratory Adjusted Analysis of Oncologic Outcomes

To account for baseline differences between cohorts, exploratory multivariable logistic regression models were constructed, adjusting for age and tumor size. The analyses were based on 9 lymph node metastasis events and 21 LVSI-positive cases. Given the limited number of outcome events relative to the number of predictors included in the models, the results should be interpreted cautiously and regarded as hypothesis-generating. In particular, the number of lymph node metastasis events was below the generally recommended threshold for stable multivariable modeling, and therefore, the estimated odds ratios should be interpreted cautiously.

After adjustment, the surgical approach was not independently associated with lymph node metastasis or lymphovascular space invasion. In contrast, tumor size remained significantly associated with both outcomes and emerged as the strongest predictor of adverse pathological features (Table 3).

### 3.5. Early Oncologic Outcomes

The median follow-up duration was 24 months in the robotic cohort and 60 months in the open-surgery cohort. During the available follow-up period, no local or distant recurrences were observed in the robotic cohort, and all patients remained disease-free at the last recorded follow-up. However, owing to the substantial difference in follow-up duration between cohorts and the absence of recurrence events in the robotic group, formal comparative survival analyses were not undertaken, and no conclusions regarding comparative oncologic efficacy can be drawn.

## 4. Discussion

This comparative cohort study provides a contemporary evaluation of robotic radical surgical management and open radical hysterectomy in patients with early-stage cervical cancer treated in the post-LACC era. The principal findings may be summarized as follows: (i) patients undergoing robotic surgery were younger and presented with significantly smaller tumors; (ii) no statistically significant differences were observed between cohorts with respect to lymph node metastasis, LVSI, or resection margin status; (iii) robotic surgery was associated with reduced blood loss and shorter hospitalization despite longer operative time; and (iv) exploratory adjusted analyses identified tumor size, rather than surgical approach, as the strongest predictor of adverse pathological features [32,33,34].

Importantly, the present findings extend our previously published prospective experience with robotic compartment-based surgery by placing these outcomes within a direct comparative framework with open radical hysterectomy. Rather than supporting a simplistic dichotomy between minimally invasive and open approaches, the results suggest that oncologic outcomes are determined by a complex interaction between tumor biology, patient selection, surgical technique, and institutional expertise [27,35].

The Laparoscopic Approach to Cervical Cancer (LACC) trial fundamentally reshaped surgical management by demonstrating inferior disease-free and overall survival following minimally invasive radical hysterectomy compared with open surgery [13,36,37].

These findings prompted substantial changes in clinical guidelines and renewed interest in identifying factors responsible for the observed survival differences. Subsequent observational studies and meta-analyses have suggested that outcomes following minimally invasive surgery may be influenced by tumor manipulation, intracorporeal colpotomy, surgeon experience, institutional volume, and patient selection rather than by surgical access alone [38,39,40,41].

Consequently, contemporary assessment of robotic surgery requires consideration of technical, biological, and patient-related factors rather than evaluation of the surgical approach in isolation.

The significantly higher lymph node yield observed in the robotic cohort should be interpreted cautiously. Although increased nodal retrieval may reflect meticulous surgical dissection, it may also be influenced by differences in pathological processing, specimen handling, institutional protocols, or reporting practices. Because the cohorts originated from different institutions and pathology departments, the observed difference cannot be attributed solely to surgical technique. Furthermore, lymph node count does not necessarily correlate with oncologic adequacy once established anatomical boundaries of lymphadenectomy have been respected.

From a biological perspective, recurrence following radical treatment of cervical cancer is increasingly understood as a consequence of occult micrometastatic disease and tumor-specific molecular characteristics rather than solely inadequate local control. Several studies have demonstrated associations between recurrence risk and established pathological factors, including LVSI, lymph node involvement, depth of stromal invasion, and tumor size [41,42,43,44,45]. Consistent with these observations, tumor size emerged as an independent predictor of lymph node metastasis and LVSI in the present study. Larger tumors are characterized by greater biological complexity, including enhanced invasive capacity, greater stromal interaction, increased intratumoral heterogeneity, and a higher likelihood of lymphovascular dissemination. In addition, accumulating evidence indicates that metastatic progression is driven by dynamic interactions between tumor cells and the surrounding microenvironment. Processes such as hypoxia-induced adaptation, angiogenesis, epithelial–mesenchymal transition, immune evasion, and tumor-derived exosomal signaling contribute to metastatic dissemination, pre-metastatic niche formation, and treatment resistance [46]. Emerging transcriptomic evidence further indicates that aberrant alternative splicing events and dysregulation of oncogenic signaling pathways, including PI3K/AKT/mTOR and TGF-β signaling, contribute to tumor progression and metastatic competence [45]. Consequently, the association between tumor size and adverse pathological features observed in the present cohort likely reflects underlying biological aggressiveness rather than merely anatomical tumor extent.

The present findings should also be interpreted in the context of the recently published SHAPE trial, which demonstrated non-inferiority of simple hysterectomy compared with radical hysterectomy in carefully selected patients with low-risk early-stage cervical cancer characterized by tumors ≤ 2 cm, limited stromal invasion, and negative preoperative nodal assessment [47,48].

Notably, the mean tumor size in the robotic cohort was 1.4 cm, suggesting that a substantial proportion of patients may have shared clinicopathological characteristics with the low-risk population represented in SHAPE. Therefore, favorable outcomes in this cohort should not be extrapolated to patients with larger tumors or higher-risk disease. Consequently, favorable outcomes observed in the robotic cohort may partly reflect treatment of biologically low-risk disease rather than the specific surgical platform or extent of radicality. Because several variables required for formal SHAPE eligibility assessment were not uniformly available, direct classification according to SHAPE criteria was not feasible. Nevertheless, the overlap between the robotic cohort and the SHAPE population should be considered when interpreting the present findings.

An additional factor that must be considered is the non-contemporaneous nature of the study cohorts. Patients in the open-surgery cohort were treated during an earlier period, whereas patients in the robotic cohort underwent surgery after publication of the LACC trial, when qualification for minimally invasive surgery became substantially more selective. Consequently, the observed differences between cohorts may reflect not only the surgical approach itself but also temporal changes in clinical practice, evolving treatment paradigms, increased awareness of tumor-spillage prevention strategies, and differences in institutional protocols. Because the cohorts were treated in different institutions and by different surgical teams, the relative contribution of these factors cannot be separated from the effects of surgical technique alone. Therefore, the present findings should be interpreted within the context of potential era-, institution-, surgeon-, and selection-related biases.

TMMR, derived from Höckel’s ontogenetic compartment theory, represents a conceptual shift in the understanding of radical hysterectomy. By targeting embryologically defined developmental compartments rather than purely anatomical landmarks, TMMR aims to achieve en bloc tumor resection while preserving autonomic function and minimizing unnecessary tissue removal. Robotic technology may facilitate the implementation of these principles through enhanced three-dimensional visualization, tremor filtration, and improved instrument articulation, thereby supporting precise dissection within mesometrial planes [49].

An additional consideration is the heterogeneity of the robotic cohort. Although the study was conceptually grounded in the implementation of robotic compartment-based surgery, not all patients underwent identical procedures. The robotic cohort included a mixture of total mesometrial resection, robotic radical hysterectomy, and, in selected cases, radical trachelectomy. Surgical tailoring was performed according to tumor characteristics and individual clinical circumstances. While this reflects real-world clinical practice, it complicates the interpretation of the results because the observed outcomes cannot be attributed exclusively to TMMR. Consequently, the present findings should be viewed as reflecting the overall performance of robotic radical surgical management rather than the efficacy of a single standardized robotic procedure.

While robotic technology may facilitate precise surgical dissection and implementation of compartment-based concepts, the present study does not permit conclusions regarding the independent contribution of either the robotic platform or TMMR to the observed outcomes. Favorable perioperative and pathological findings in the robotic cohort may have been influenced by stricter patient selection, smaller tumor burden, favorable tumor biology, and adherence to contemporary oncologic principles designed to minimize tumor exposure and manipulation. Importantly, exploratory adjusted analyses identified tumor size, rather than surgical approach, as the principal predictor of lymph node metastasis and LVSI. Therefore, the present findings should not be interpreted as evidence of oncologic superiority or equivalence of robotic surgery compared with open radical hysterectomy [50,51,52].

Another important factor that may have contributed to the favorable short-term outcomes observed in the robotic cohort is the systematic application of tumor-exposure prevention measures. Robotic procedures were performed without conventional transuterine uterine manipulators and utilized a MyTube^®^ vacuum retractor that did not enter the uterine cavity or traverse the endocervical canal. These precautions are consistent with contemporary tumor-isolation strategies and previously reported recommendations aimed at minimizing tumor spillage during minimally invasive radical hysterectomy [53,54]. However, the present findings do not allow the independent contribution of these measures to be separated from the effects of patient selection, tumor characteristics, surgeon expertise, or institutional factors.

Beyond tumor biology, technical factors and institutional expertise may also influence oncologic outcomes. Previous post-LACC analyses have highlighted the potential impact of tumor manipulation, intracorporeal colpotomy, surgeon experience, and institutional volume on recurrence risk [26,55,56,57]. In the present study, robotic procedures were performed by a specialized surgical team experienced in robotic and compartment-based surgery, using standardized measures intended to minimize tumor manipulation. Importantly, robotic procedures were performed by board-certified, robotically certified gynecologic oncologists. Nevertheless, because the robotic cohort included only 20 patients and formal survival comparisons could not be performed, the absence of recurrence during follow-up should be interpreted as a preliminary observation rather than evidence of oncologic equivalence. Validation in larger prospective cohorts with longer follow-up and appropriately matched comparator groups remains necessary [58,59,60].

Several limitations of this study should be acknowledged. First, the retrospective comparative design, small sample size, and limited number of oncologic events restricted statistical power and increased the risk of type II error. Consequently, the absence of statistically significant differences between groups should not be interpreted as evidence of oncologic equivalence. Second, important baseline imbalances were present between cohorts, particularly with respect to patient age and tumor size, reflecting non-randomized patient selection and changes in clinical practice over time. Third, the robotic and open cohorts originated from different institutions and were treated by different surgical teams during different treatment periods, introducing potential era-, institution-, and surgeon-related biases that could not be fully controlled. Fourth, the robotic cohort was heterogeneous and included TMMR, robotic radical hysterectomy, and radical trachelectomy, limiting procedure-specific interpretation. Finally, follow-up duration differed between cohorts, precluding robust comparative survival analyses.

Despite these limitations, the present study provides clinically relevant insights into robotic radical surgical management in the post-LACC era. The findings suggest that pathological outcomes may be influenced more strongly by intrinsic tumor characteristics, particularly tumor size, than by surgical approach alone. At the same time, they highlight the potential importance of careful patient selection, tumor-exposure prevention strategies, and surgical expertise in achieving favorable outcomes.

More broadly, these observations support the growing recognition that successful management of cervical cancer requires the integration of surgical excellence with a deeper understanding of tumor biology. Future studies evaluating robotic and compartment-based surgical approaches should incorporate molecular, pathological, and translational biomarkers capable of refining recurrence-risk stratification and identifying patients most likely to benefit from specific surgical strategies. Larger prospective investigations with balanced cohorts, standardized operative protocols, and long-term follow-up are required before definitive conclusions regarding the comparative oncologic effectiveness of robotic and open surgery can be established.

## 5. Conclusions

In this exploratory comparative cohort study, robotic radical surgical management, including selected compartment-based procedures, appeared feasible in carefully selected patients with early-stage cervical cancer and was associated with favorable short-term perioperative outcomes, including reduced blood loss and shorter hospitalization. No statistically significant differences in pathological parameters, including lymph node metastasis, lymphovascular space invasion, or resection margin status, were observed between the robotic and open-surgery cohorts.

However, the robotic cohort was characterized by younger age, smaller tumor size, and more selective patient qualification. Exploratory adjusted analyses identified tumor size, rather than surgical approach, as the principal predictor of lymph node metastasis and lymphovascular space invasion, highlighting the importance of underlying tumor biology in determining pathological risk.

Given the retrospective design, small sample size, limited number of outcome events, baseline imbalances between cohorts, heterogeneity of robotic procedures, methodological differences in tumor-size assessment, and the non-contemporaneous nature of the study groups, the present findings should be interpreted with caution. The favorable outcomes observed in the robotic cohort may reflect strict patient selection, lower tumor burden, tumor-exposure prevention strategies, high surgeon expertise and procedural standardization, and institutional factors rather than the independent effect of the robotic platform or compartment-based surgery. Accordingly, this study does not establish oncologic equivalence between robotic radical surgery and open radical hysterectomy. Larger prospective studies with balanced cohorts, standardized surgical protocols, comprehensive assessment of tumor biology, and long-term follow-up are required to clarify the relative contribution of surgical approach, patient selection, and biological factors to oncologic outcomes.

## Figures and Tables

**Table 1:** Baseline demographic and clinical characteristics of patients undergoing robotic and open radical surgery for early-stage cervical cancer.

Parameter	Robotic Surgery (n = 20)	Open Surgery (Laparotomy Group) (n = 22)	*p*-Value
Number of patients	20	22	—
Age (years), mean ± SD	45.67 ± 9.31	56.23 ± 15.87	0.012 †
Body weight (kg), mean ± SD	70.90 ± 16.59	69.80 ± 14.72	0.210
Height (cm), mean ± SD	167.19 ± 6.11	167.81 ±5.34	0.187
Body mass index (kg/m^2^), mean ± SD	25.18 ± 5.18	29.54 ± 8.36 *	0.137 †
HPV infection, n (%)	4 (20.0%)	8 (36.36%)	0.28
HSV infection, n (%)	2 (10.0%)	1 (4.54%)	0.59
Colposcopy performed, n (%)	20 (100%)	22 (100%)	—
Diagnostic conization prior to surgery, n (%)	8 (40.0%)	3 (13.60%)	0.08
Tumor size (cm), mean ± SD (range)	1.4 ± 0.7 (0.1–2.5)	3.07 ± 1.1 (0.1–4.0)	0.003 †

* Value marked due to higher dispersion within the open surgery group. † *p*-values were calculated using Student’s *t*-test for continuous variables; categorical variables were analyzed using the χ^2^ test or Fisher’s exact test, as appropriate. — not applicable (no statistical comparison performed due to constant values across groups).

**Table 2:** Comparative perioperative and oncologic outcomes of robotic and open radical surgery in early-stage cervical cancer.

Parameter	Robotic Surgery (n = 20)	Open Surgery (n = 22)	*p*-Value
Tumor size (cm), mean ± SD	1.4 ± 0.7	3.07 ± 1.1	0.003
Lymph nodes retrieved, median (range)	18 (12–27)	9 (3–26)	<0.001
Lymph node metastases (pN1), n (%)	4 (20.0%)	5 (22.7%)	1.000
LVSI positivity, n (%)	7 (33.3%)	14 (63.6%)	0.121
R0 resection, n (%)	20 (100%)	21 (95.5%)	1.000
Operative time (minutes)	178 ± 42	150 ± 38	0.028
Blood loss (mL)	112 ± 61	518 ± 98	<0.001
Mean hospital stay (days)	4.2 ± 1.60	6.2 ± 1.40	<0.001

Continuous variables are presented as mean ± SD or median (range), depending on distribution normality, and were compared using Student’s *t*-test or the Mann–Whitney U test, as appropriate. Categorical variables are expressed as counts and percentages and were analyzed using Fisher’s exact test. A two-sided *p*-value < 0.05 was considered statistically significant.

**Table 3:** Exploratory multivariable regression analysis of factors associated with adverse pathological features in early-stage cervical cancer.

Outcome	Variable	Adjusted OR	95% CI	*p*-Value
Lymph node metastasis (pN1)	Surgical approach (robotic vs. open)	0.92	0.18–4.65	0.92
	Age (per year)	1.01	0.97–1.05	0.64
	Tumor size (per cm)	1.78	1.05–3.10	0.032
Lymphovascular space invasion (LVSI)	Surgical approach (robotic vs. open)	0.54	0.16–1.87	0.33
	Age (per year)	1.02	0.98–1.06	0.31
	Tumor size (per cm)	2.12	1.12–4.02	0.021

Adjusted OR, adjusted odds ratio; CI, confidence interval. Model for lymph node metastasis based on 9 events; model for LVSI based on 21 events.

## Data Availability

The data presented in this study are available upon reasonable request from the corresponding author. The data are not publicly available due to ethical and privacy restrictions related to patient confidentiality.

## Abbreviations

The following abbreviations are used in this manuscript:

BMI	body mass index
CI	confidence interval
DFS	disease-free survival
FIGO	International Federation of Gynecology and Obstetrics
HPV	human papillomavirus
HSV	herpes simplex virus
LACC	laparoscopic approach to cervical cancer
LVSI	lymphovascular space invasion
MRI	magnetic resonance imaging
OR	odds ratio
pN1	pathological lymph node metastasis
R0	microscopically negative resection margin
R1	microscopically positive resection margin
SD	standard deviation
TMMR	total mesometrial resection
